# Supplementary material for: In-situ direct seawater electrolysis using floating platform in ocean with uncontrollable wave motion
Source: Nat Commun. 2024 Jun 21;15:5305. doi: 10.1038/s41467-024-49639-6 (PMC11192878; doi:10.1038/s41467-024-49639-6)
Supplement: Supplementary file 1 — Supplementary Information [file 41467_2024_49639_MOESM1_ESM.pdf]

Supplementary Information

In-situ direct seawater electrolysis using floating platform in ocean with uncontrollable wave motion

Tao Liu✉[1,2,3,10], Zhiyu Zhao[1,2,4,10], Wenbin Tang[5,6,10], Yi Chen[7], Cheng Lan[1,2,4], Liangyu Zhu[2], Wenchuan Jiang[1,2,4], Yifan Wu[1,2,4], Yunpeng Wang[4,6], Zezhou Yang[2], Dongsheng Yang[4,5], Qijun Wang[8], Lunbo Luo[9], Taisheng Liu✉[7], Heping Xie✉[1,2,3,4]

1. State Key Laboratory of Intelligent Construction and Healthy Operation and Maintenance of Deep Underground Engineering, Shenzhen University & Sichuan University, Shenzhen 518060, China.
2. Institute of New Energy and Low-Carbon Technology, Sichuan University, Chengdu 610065, China.
3. Guangdong Provincial Key Laboratory of Deep Earth Sciences and Geothermal Energy Exploitation and Utilization, College of Civil and Transportation Engineering, Shenzhen University, Shenzhen 518060, China.
4. Shenzhen Key Laboratory of Deep Engineering Science and Green Energy, Institute of Deep Earth Sciences and Green Energy, Shenzhen University, Shenzhen 518060, China.
5. College of Polymer Science and Engineering, Sichuan University, Chengdu 610065, China.
6. School of Chemical Engineering, Sichuan University, Chengdu 610065, China.
7. Dongfang Electric (Fujian) Innovation Institute Co., Ltd, Fuzhou 350108, China.
8. Dongfang Electric Wind Power Co., Ltd. Deyang 618000, China.
9. Fujian Branch, China Three Gorges Corporation, Fuzhou 350014, China.
10. These authors contributed equally: Tao Liu, Zhiyu Zhao, Wenbin Tang

Correspondence should be addressed to Heping Xie：xiehp@scu.edu.cn.

Tao Liu: liutao3200023@scu.edu.cn.

Taisheng Liu: liuts@dongfang.com.

The PDF file includes:

Title
Author information
Table of Contents
Supplementary Figures 1 to 16
References 1 to 2

Table of Contents

Supplementary Figure 1 – Water migration behaviour at simulated sea wave 3

Supplementary Figure 2 – 500-h durability test in the laboratory 4

Supplementary Figure 3 – Seawater electrolyser and condenser 5

Supplementary Figure 4 – COMSOL simulation of seawater electrolyser 6

Supplementary Figure 5 – Photos of anchor 7

Supplementary Figure 6 – Photo of removed water in the ambience of platform 8

Supplementary Figure 7 – Wind turbine network 9

Supplementary Figure 8 – Stabilizing effect of uninterruptible power supply 10

Supplementary Figure 9 – Simulation of floating platform 11

Supplementary Figure 10 – Photo of cable 12

Supplementary Figure 11 – Stability test of seawater electrolyser in ocean 13

Supplementary Figure 12 – LSV of seawater electrolysers operated in ocean 14

Supplementary Figure 13 – Hydrogen purity 15

Supplementary Figure 14 – Ion concentration 16

Supplementary Figure 15 – SDE concentration 17

Supplementary Figure 16 – Optimization and outlook 18

Supplementary references 19

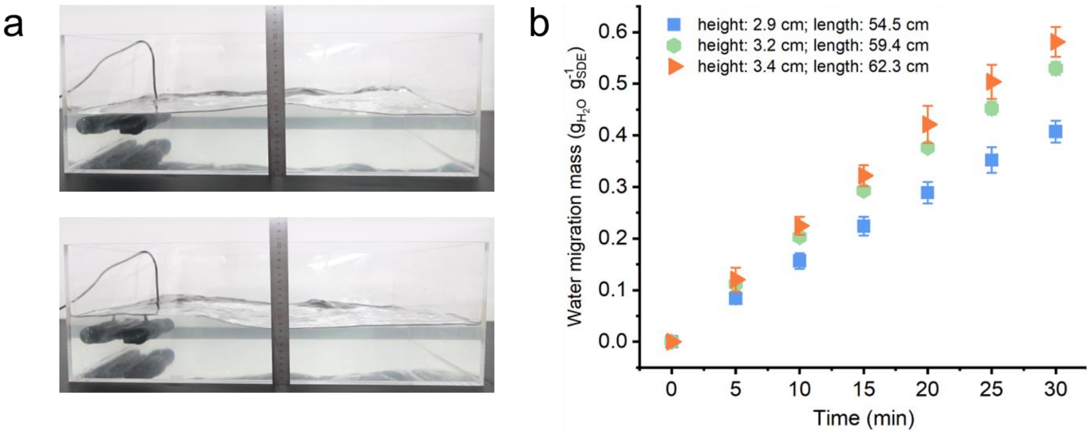

Supplementary Figure 1 – Water migration behaviour at simulated sea wave. a, The photo of wave heights. **b,** The water migration mass various with time at different wave heights. All error bars indicate the standard deviation at three measurements.

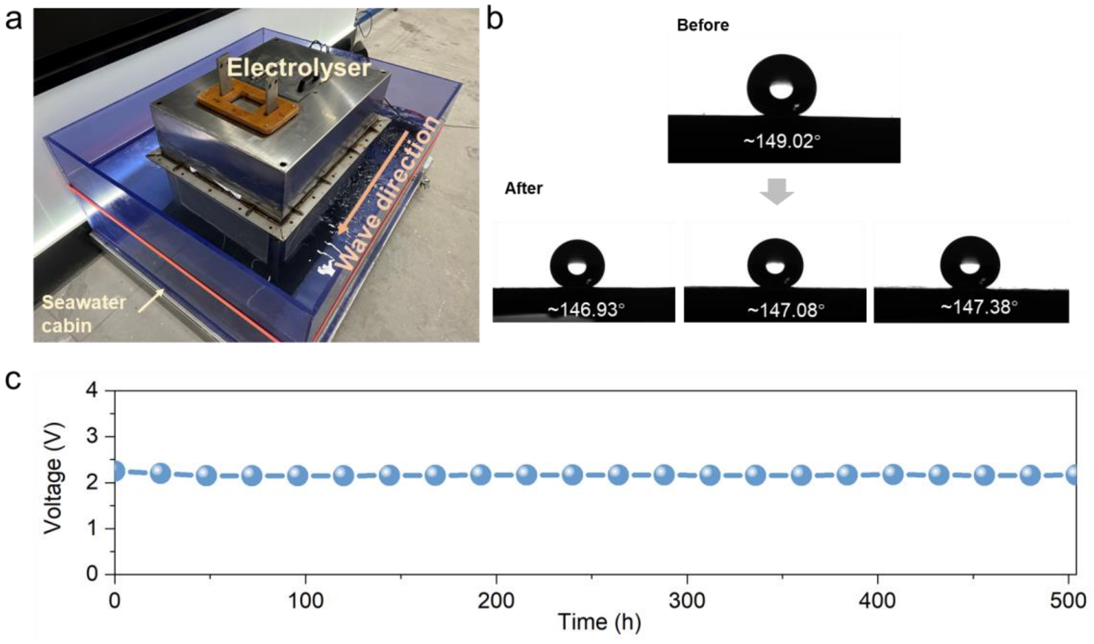

Supplementary Figure 2 – 500-h durability test in the laboratory. a, Photograph of the seawater electrolyser testing in the seawater cabin with simulated wave motion environment. **b**, Contact angle of PTFE membrane before and after test. **c**, Stability curve of electrolysis.

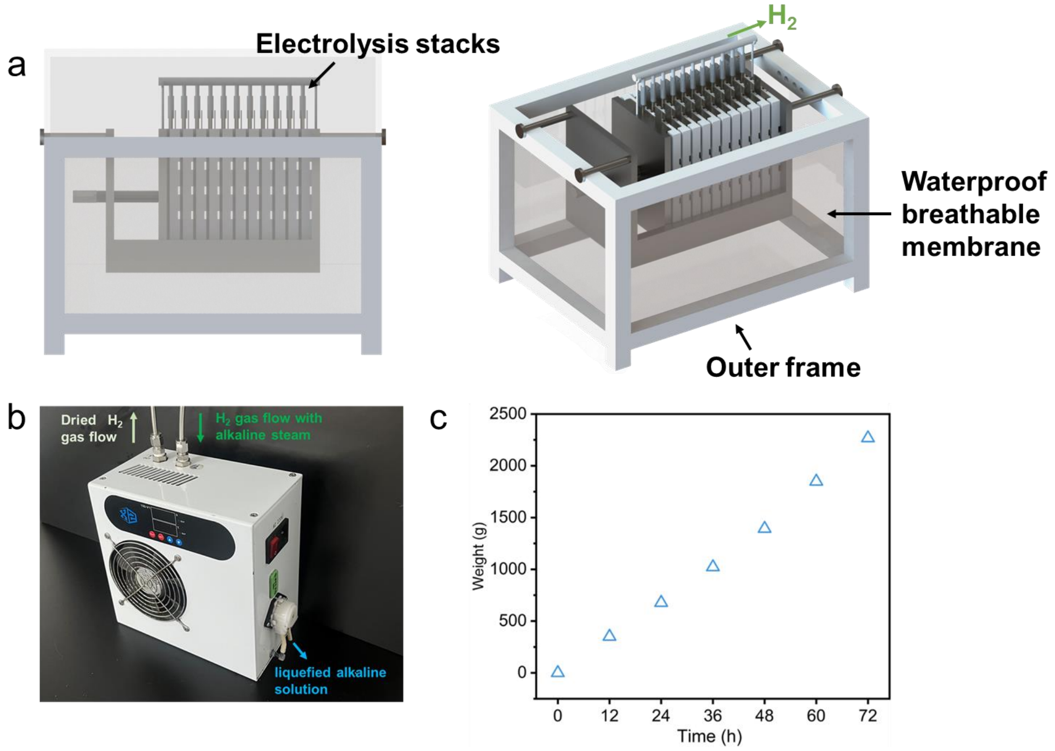

57

58 **Supplementary Figure 3 – Seawater electrolyser and condenser. a,** Diagram of seawater electrolyser. **b,** Photo of condenser. **c,** Weight of condensed solution various with time.

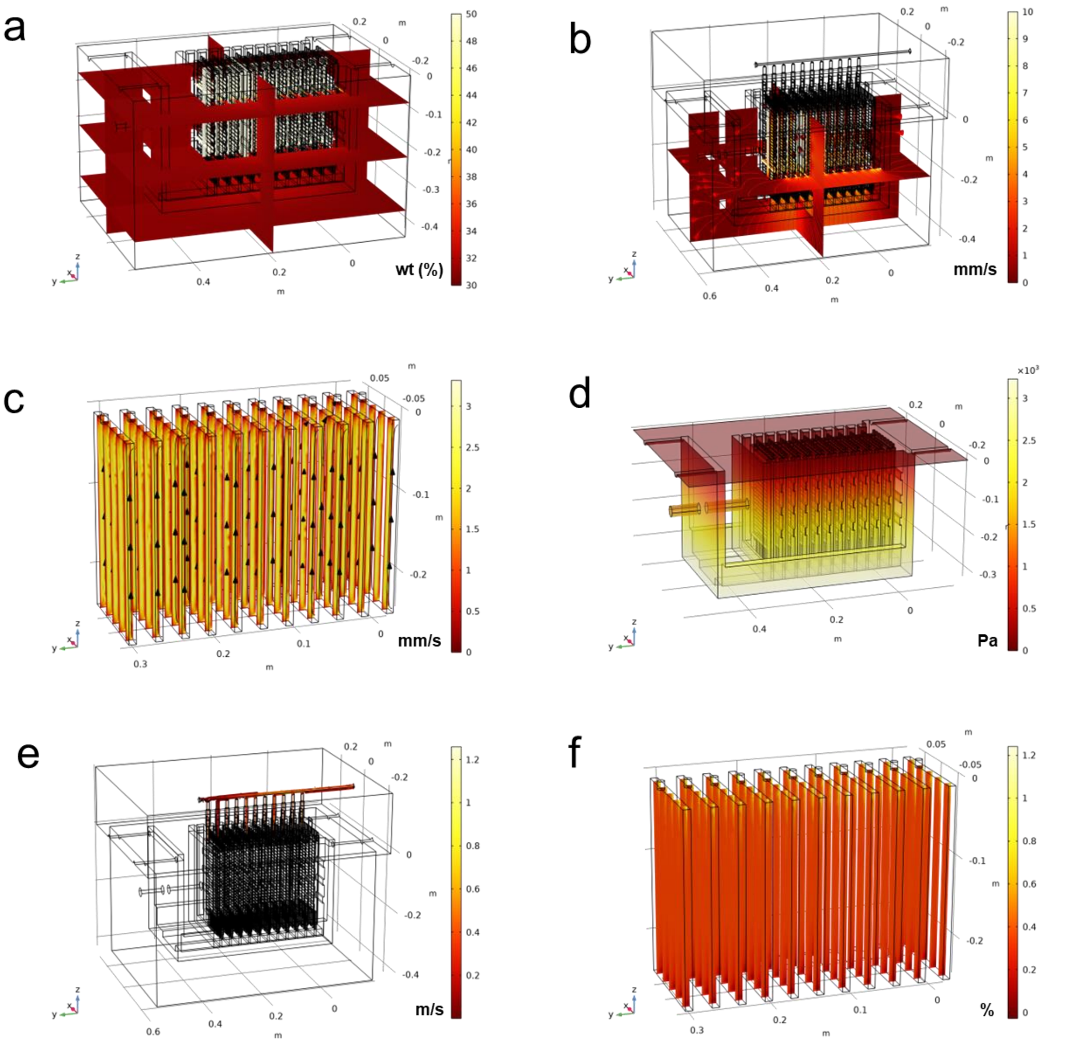

Supplementary Figure 4 - COMSOL simulation of seawater electrolyser. a, Distribution of KOH electrolyte concentration surrounding stacks. **b,** The flow rate distribution of KOH electrolyte. **c,** The exchange of electrolyte inside and out of the stacks. **d,** The pressure distribution of the stacks. **e,** Gas distribution in the cathode cavity. **f**, Gas distribution in cathode.

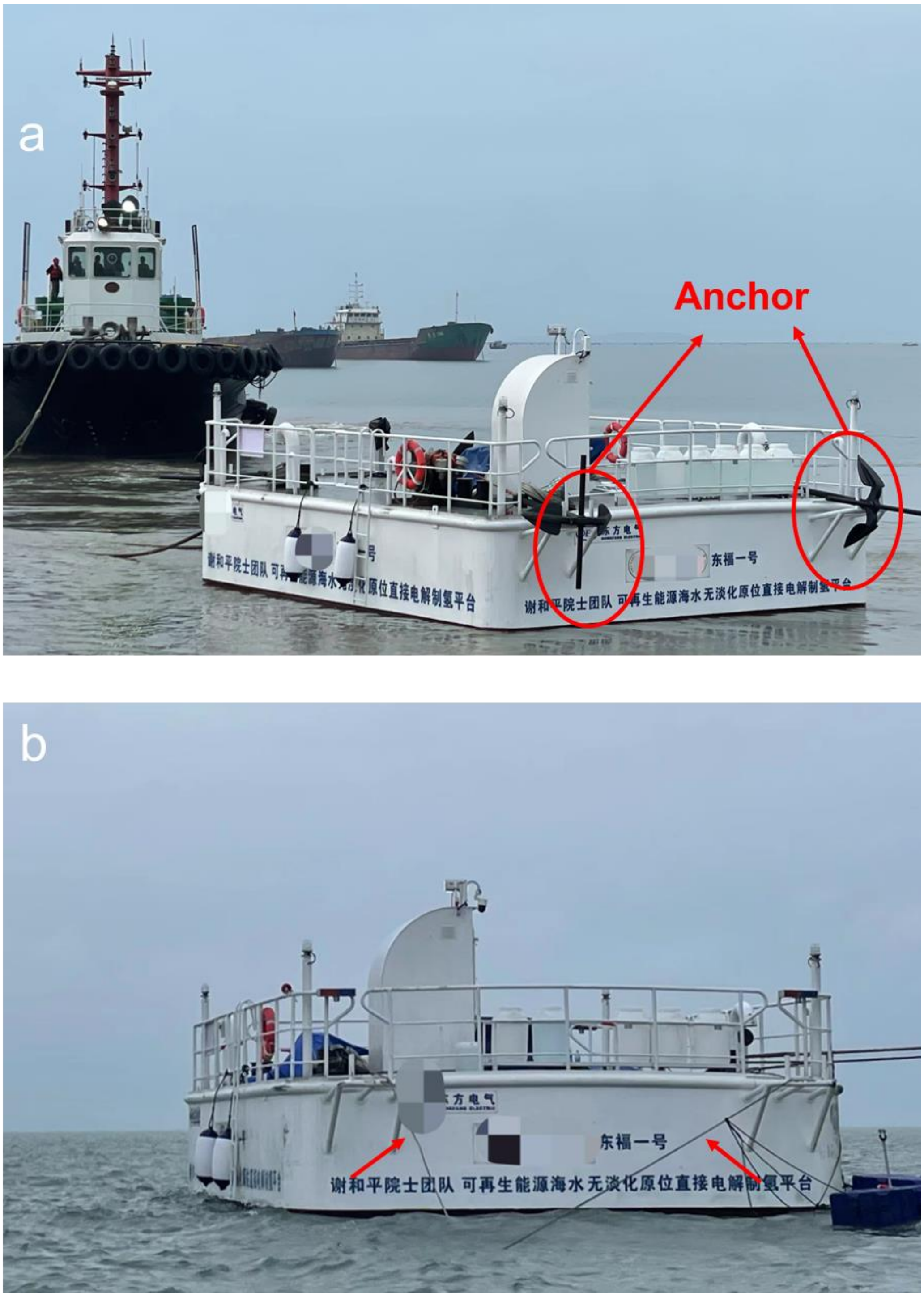

Supplementary Figure 5 – Photos of anchor. Anchors are fixed to the seabed gravel to prevent the floating body from being dragged. The depth of the experimental sea area is approximately 10 ~ 20 m.

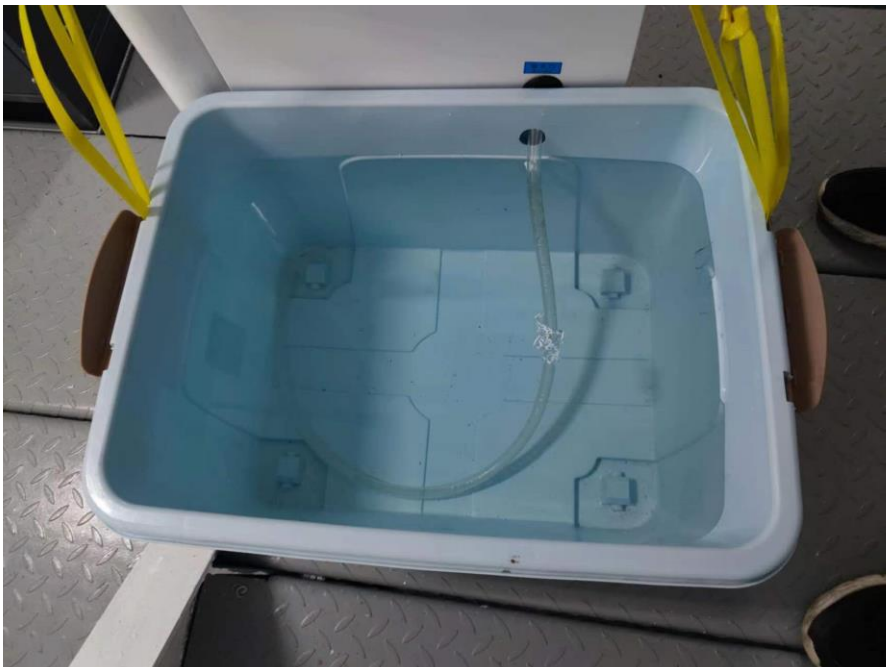

Supplementary Figure 6 – Photo of removed water in the ambience of platform.

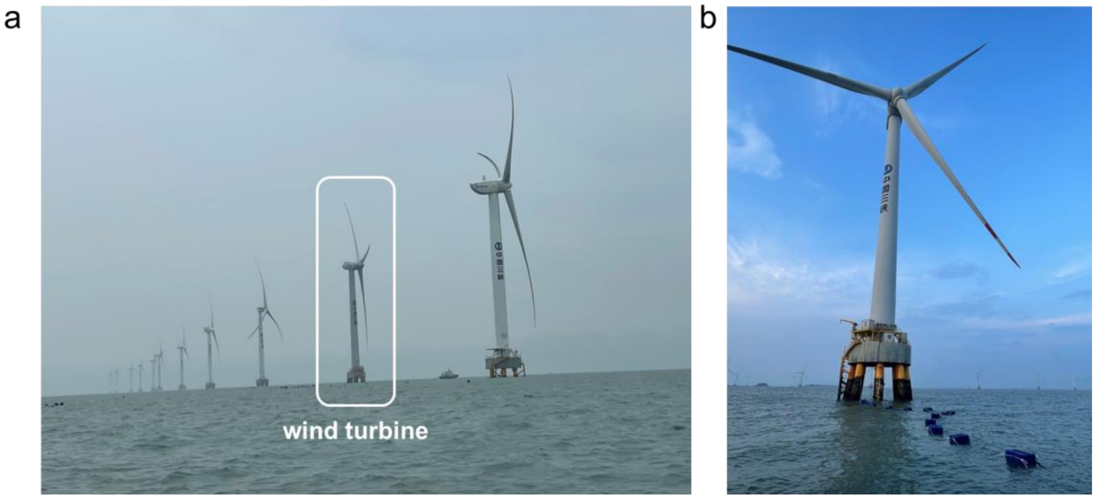

Supplementary Figure 7 – Wind turbine network. a, Wind turbine network was sited at the Xinghua Bay, Fujian Province. When one of them has insufficient power output, the electricity generated by other interconnected wind turbines will supplement it, forming a smart allocation network. **b,** offshore wind turbine with 10 MW power.

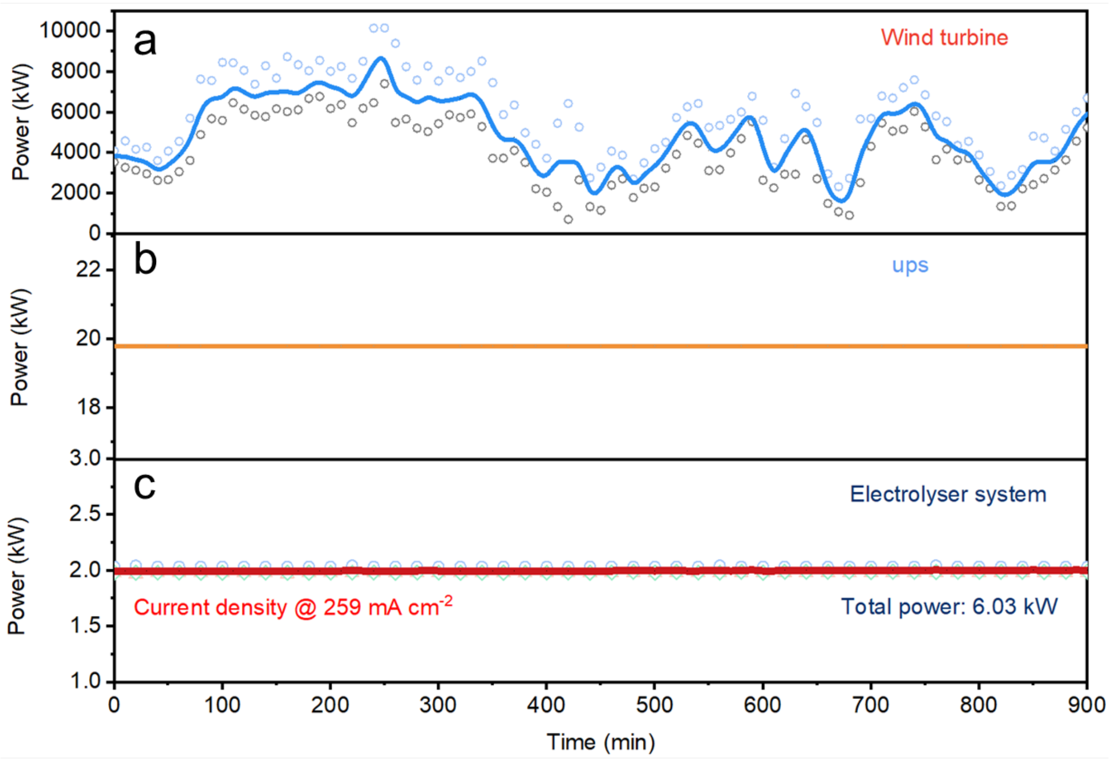

Supplementary Figure 8 - Stabilizing effect of uninterruptible power supply. a, The fluctuation of turbine power various with wind speed. **b**, UPS stably outputs approximately 19.8 kW power. **c,** Total power of electrolyser system was approximately 6.03 kW at current density of 259 mA cm^{-2}.

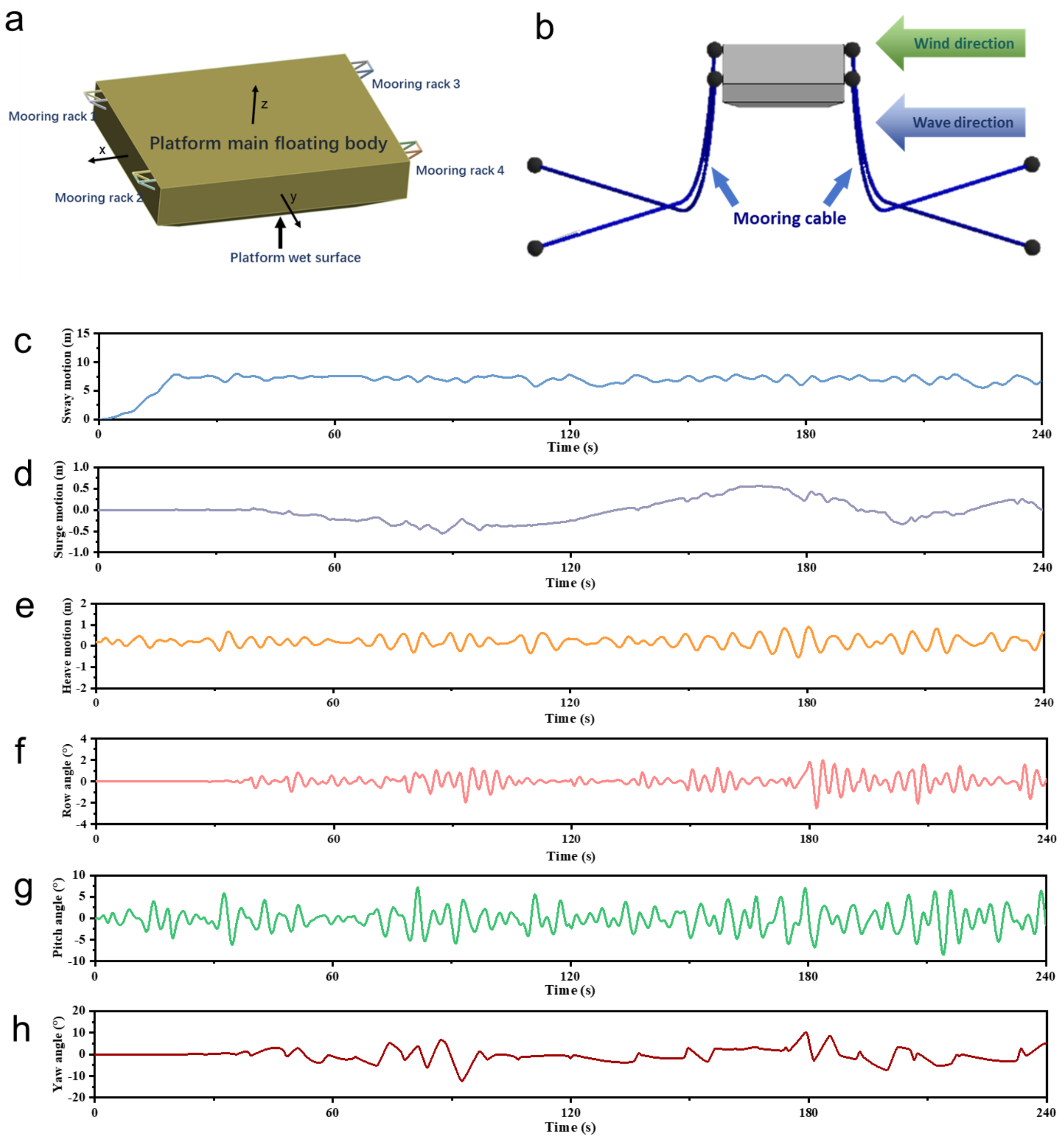

Supplementary Figure 9 – Simulation of floating platform[1-2]. a, Floating platform model. **b,** Simulation conditions. Motion distance in the X direction (sway) (**c**), Y direction (surge) (**d**) and Z direction (heave) (**e**). Deviation angle in X direction (row) (**f**), Y direction (pitch) (**g**) and Z direction (yaw) (**h**).

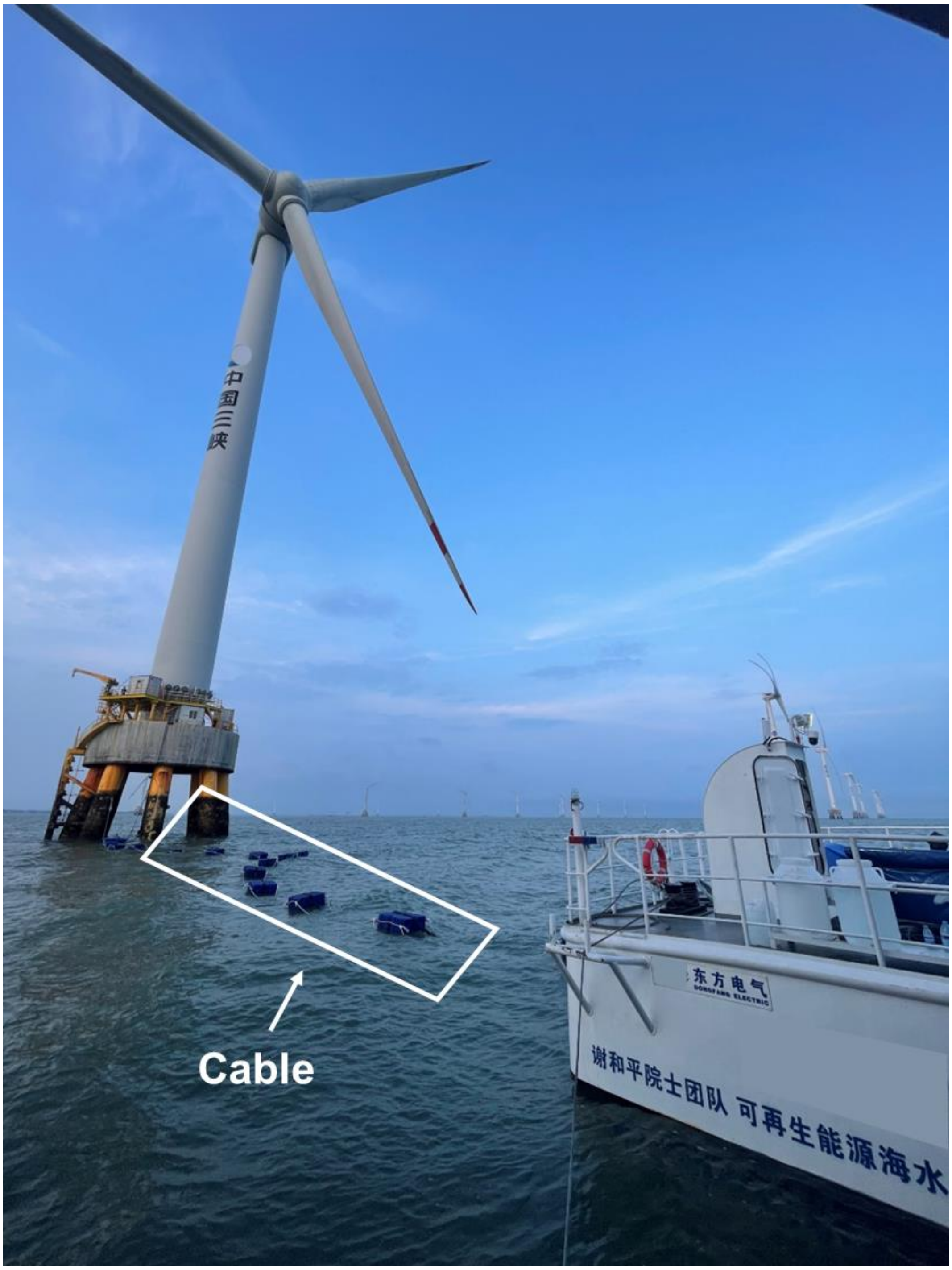

Supplementary Figure 10 – Photo of cable. The photo of cable connected with wind turbine and floating electrolysis system.

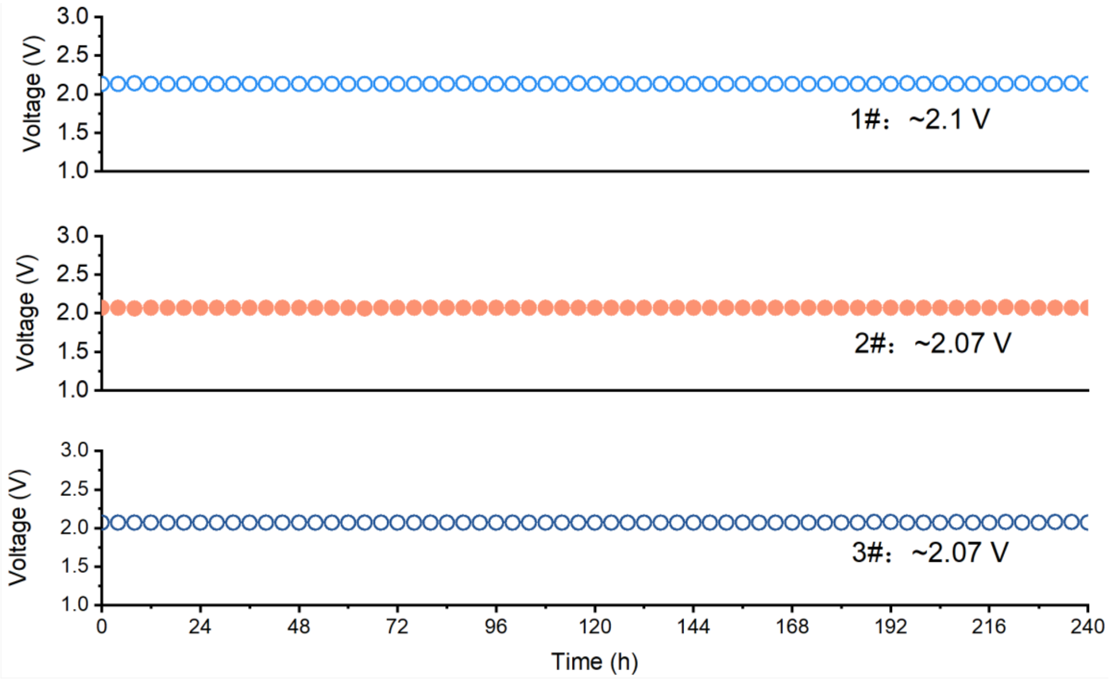

Supplementary Figure 11 – Stability test of seawater electrolyser in ocean. The stability of three electrolysers module at Xinghua Bay seawater with uncontrollable fluctuating environment. The 1#, 2# and 3# electrolyser all exhibited excellent stability of over 240 h on the fluctuating seawater at approximately 2.1 V, 2.07 V and 2.07 V voltage.

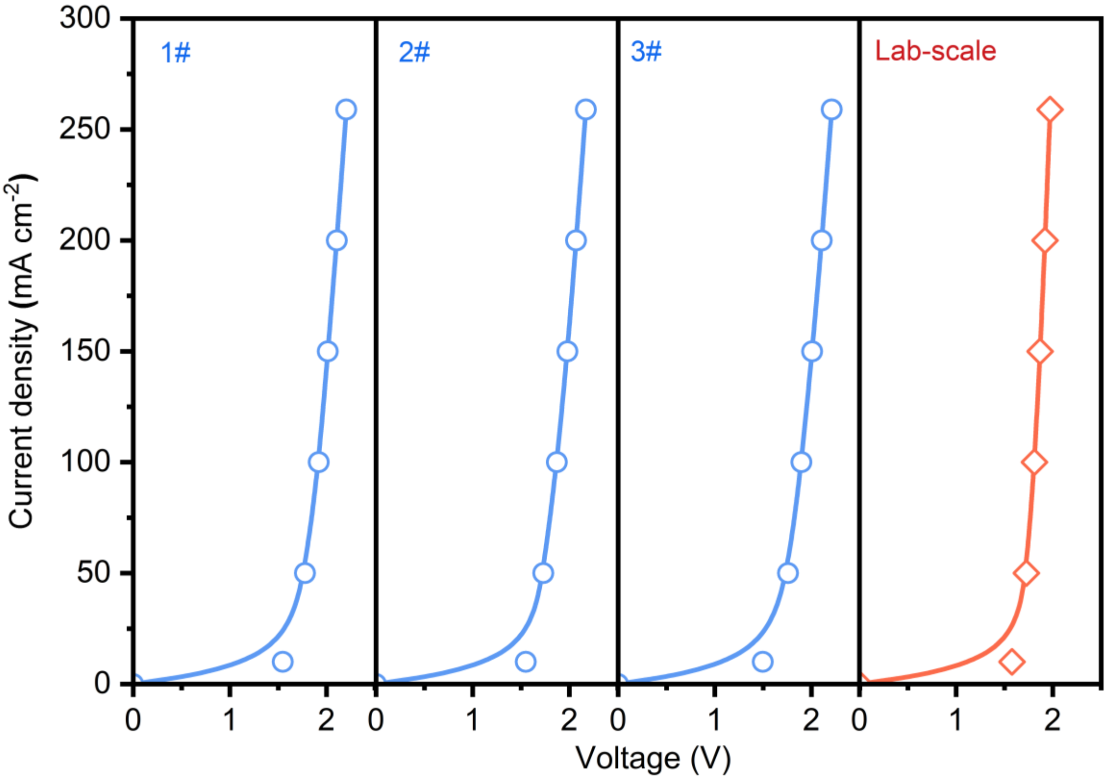

Supplementary Figure 12 - LSV of seawater electrolysers operated in ocean. The voltage of 1#, 2# and 3# electrolyser operated in ocean is 2.14 V, 2.12 V, and 2.14 V at 250 mA cm^{-2} current density, which is similar as the voltage of lab-scale electrolyser at the same current density.

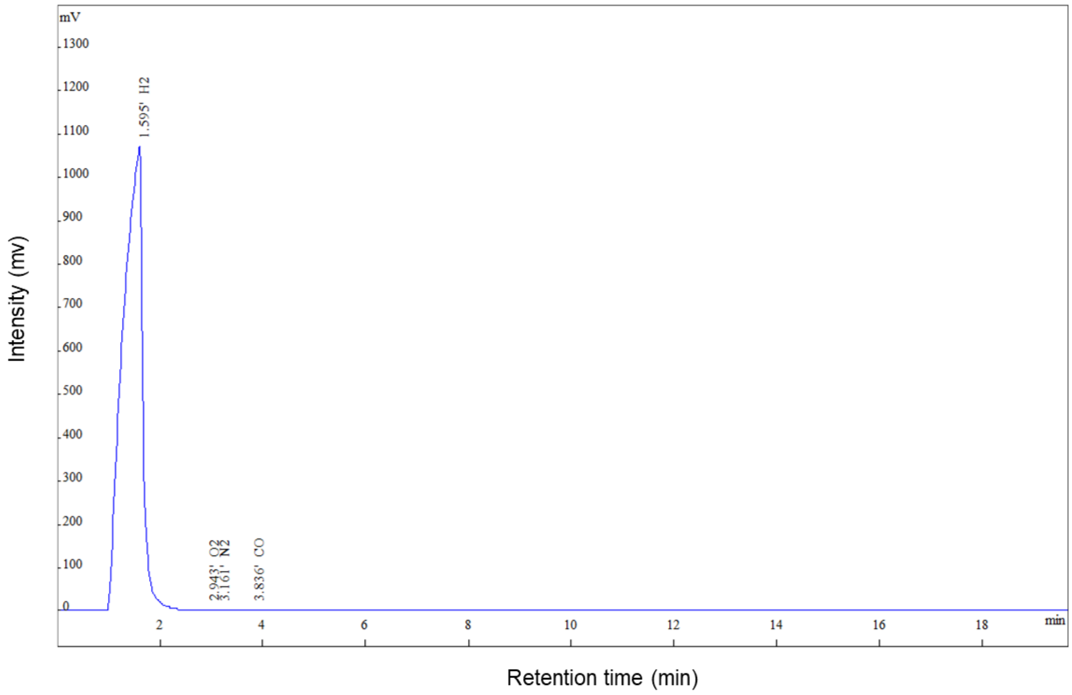

Supplementary Figure 13 – Hydrogen purity. The hydrogen purity reaches over 99.9%.

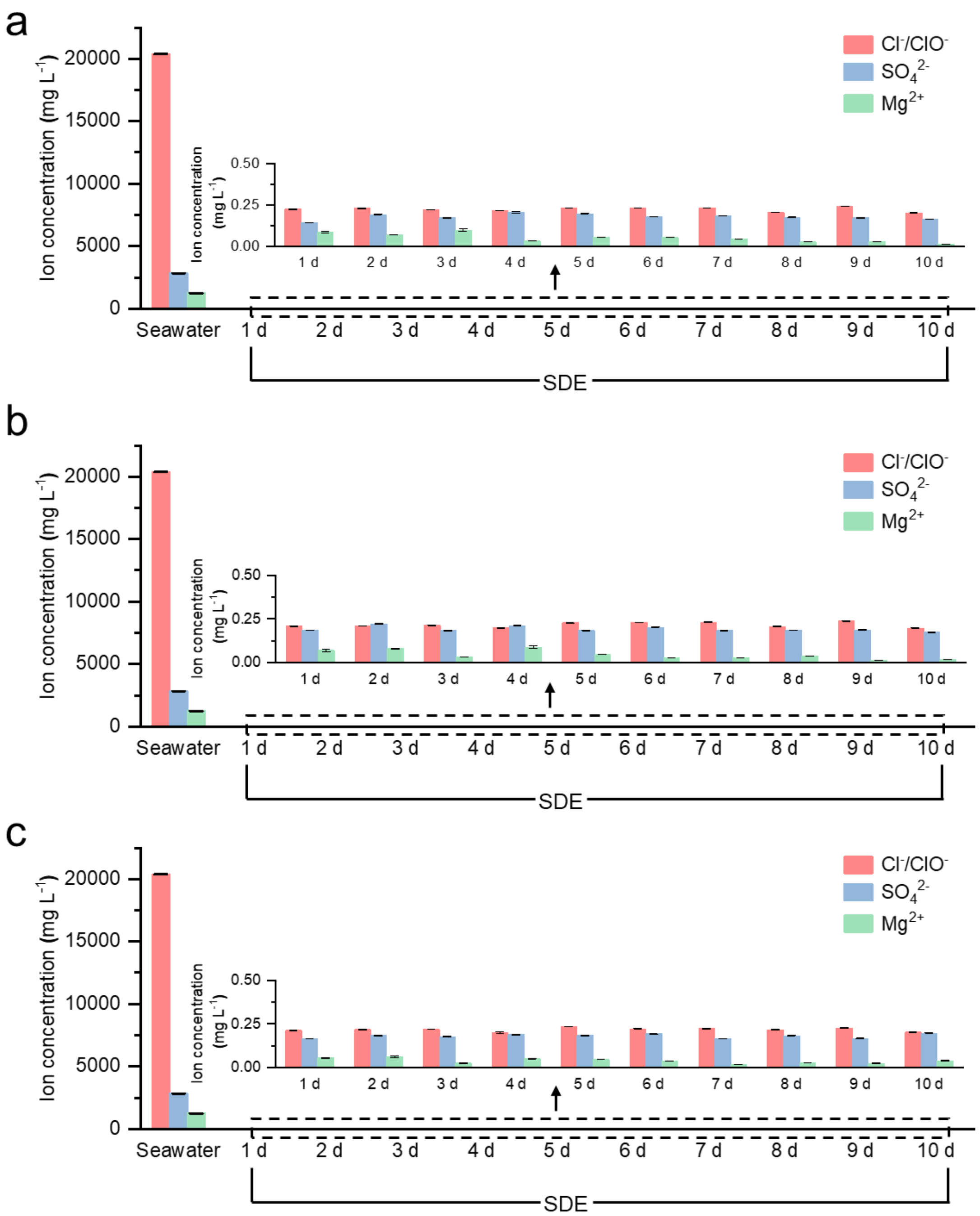

Supplementary Figure 14 – Ion concentration. The concentration of seawater electrolyser 1# (**a**), 2# (**b**) and 3# (**c**) during offshore testing. All error bars indicate the standard deviation at three measurements.

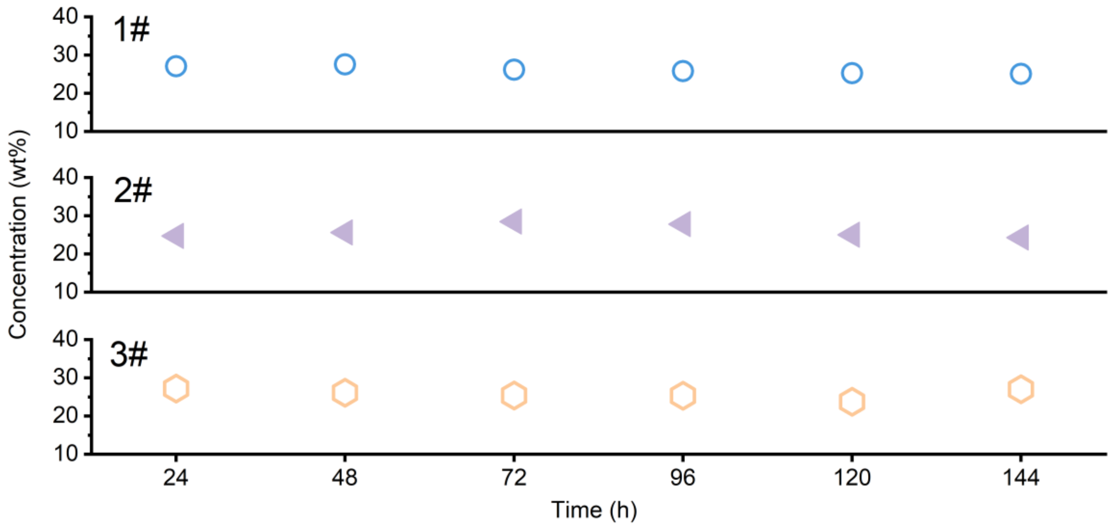

Supplementary Figure 15 – SDE concentration. We have measured the OH^- concentration in SDE. The OH^- concentration maintained 25 wt% ~ 28 wt% during the electrolysis operation in uncontrollable fluctuating environment.

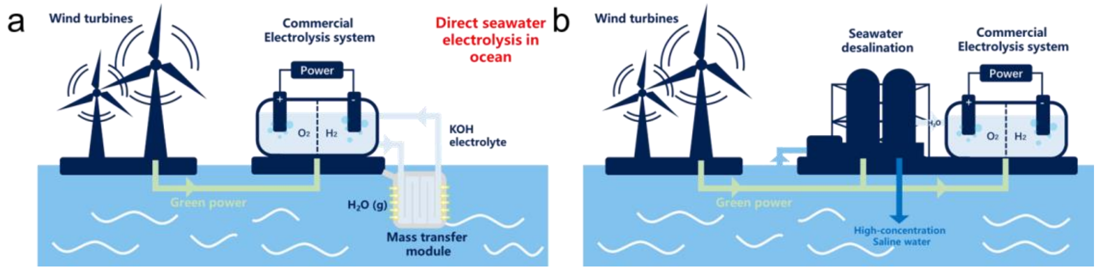

Supplementary Figure 16 – Optimization and outlook. a, Split type of in situ direct seawater electrolysis. **b**, Water electrolysis with seawater desalination.

Supplementary References

1. Journe J M J, Massie W W. Offshore hydromechanics. Delft University of Technology, (2001).
2. Chakrabarti S. Handbook of offshore engineering (2-volume set). Elsevier, (2005).
